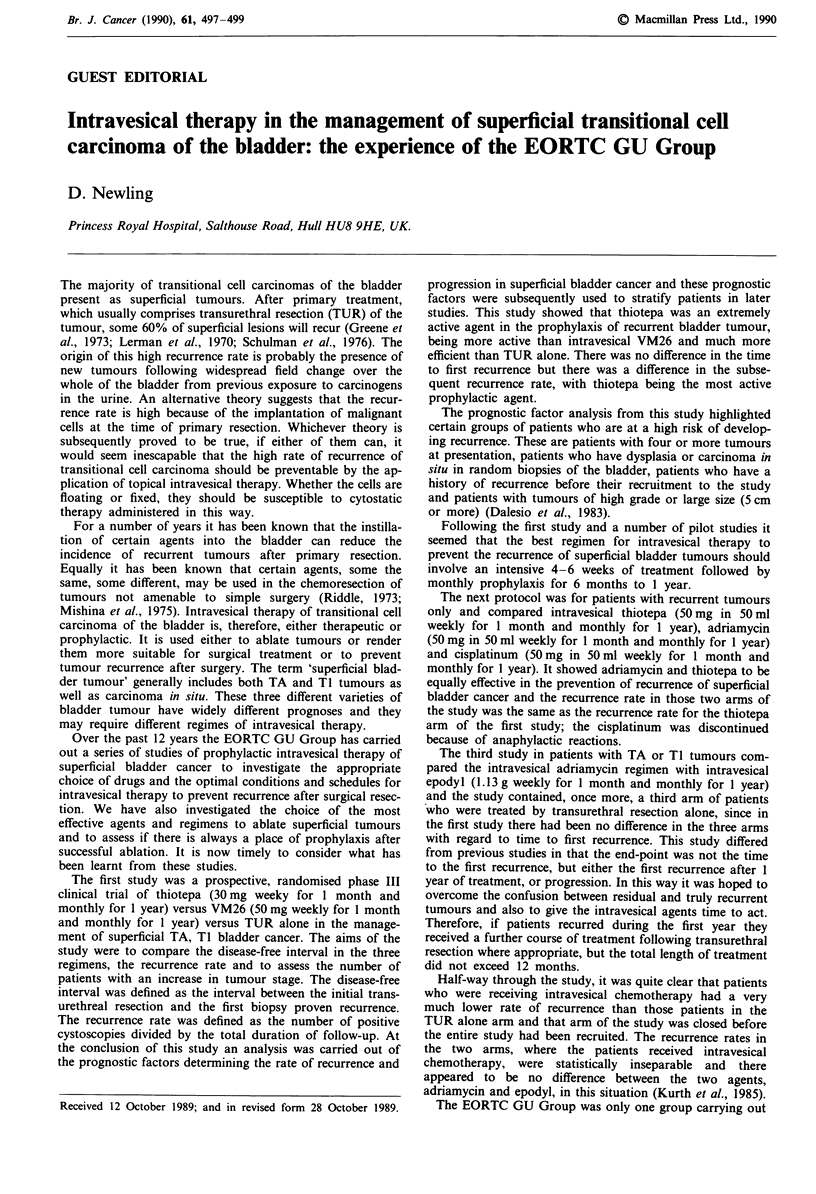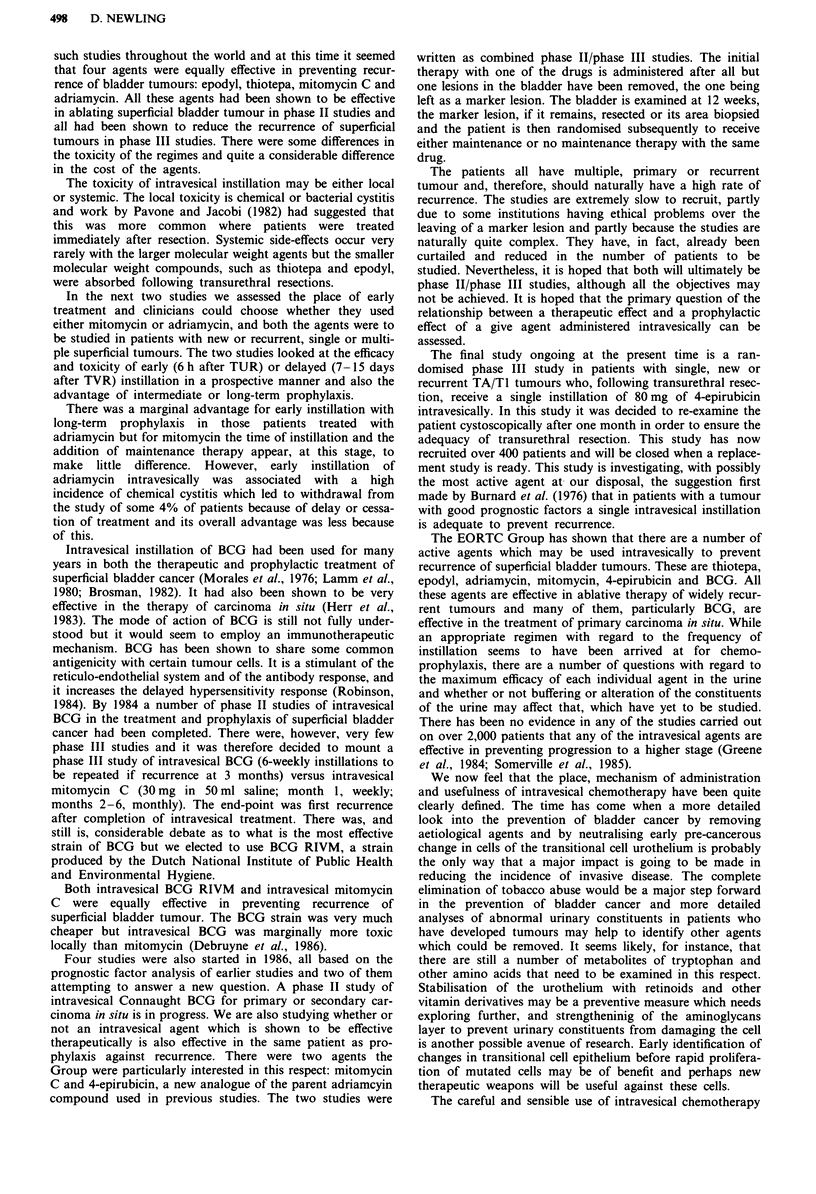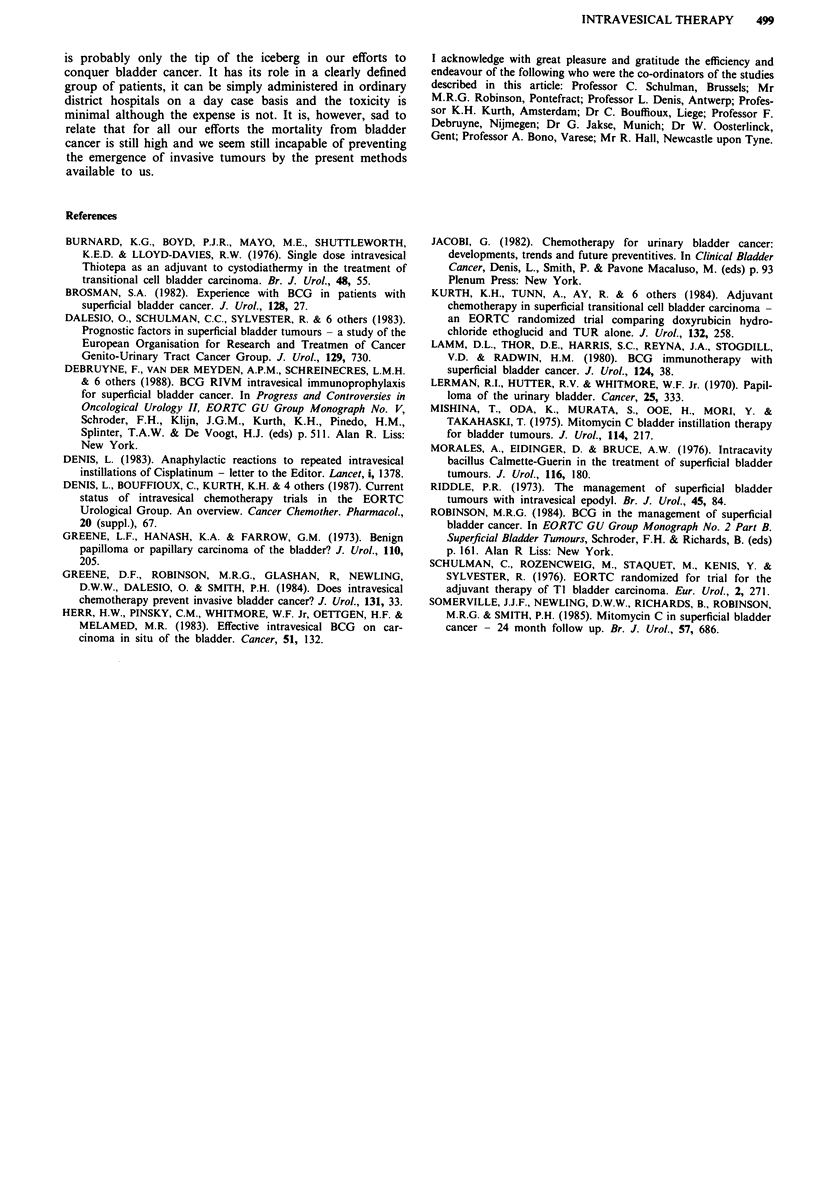# Intravesical therapy in the management of superficial transitional cell carcinoma of the bladder: the experience of the EORTC GU Group.

**DOI:** 10.1038/bjc.1990.112

**Published:** 1990-04

**Authors:** D. Newling

**Affiliations:** Princess Royal Hospital, Hull, UK.


Br. J. Cancer (1990), 61, 497–499 © Macmillan Press Ltd., 1990

GUEST EDITORIAL

### Intravesical therapy in the management of superficial transitional cell carcinoma of the bladder: the experience of the EORTC GU Group

D. Newling

*Princess Royal Hospital, Salthouse Road, Hull HU8 9HE, UK.*

The majority of transitional cell carcinomas of the bladder present as superficial tumours. After primary treatment, which usually comprises transurethral resection (TUR) of the tumour, some 60% of superficial lesions will recur (Greene *et al.*, 1973; Lerman *et al.*, 1970; Schulman *et al.*, 1976). The origin of this high recurrence rate is probably the presence of new tumours following widespread field change over the whole of the bladder from previous exposure to carcinogens in the urine. An alternative theory suggests that the recurrence rate is high because of the implantation of malignant cells at the time of primary resection. Whichever theory is subsequently proved to be true, if either of them can, it would seem inescapable that the high rate of recurrence of transitional cell carcinoma should be preventable by the application of topical intravesical therapy. Whether the cells are floating or fixed, they should be susceptible to cytostatic therapy administered in this way.

For a number of years it has been known that the instillation of certain agents into the bladder can reduce the incidence of recurrent tumours after primary resection. Equally it has been known that certain agents, some the same, some different, may be used in the chemoresection of tumours not amenable to simple surgery (Riddle, 1973; Mishina *et al.*, 1975). Intravesical therapy of transitional cell carcinoma of the bladder is, therefore, either therapeutic or prophylactic. It is used either to ablate tumours or render them more suitable for surgical treatment or to prevent tumour recurrence after surgery. The term 'superficial bladder tumour' generally includes both TA and T1 tumours as well as carcinoma *in situ*. These three different varieties of bladder tumour have widely different prognoses and they may require different regimes of intravesical therapy.

Over the past 12 years the EORTC GU Group has carried out a series of studies of prophylactic intravesical therapy of superficial bladder cancer to investigate the appropriate choice of drugs and the optimal conditions and schedules for intravesical therapy to prevent recurrence after surgical resection. We have also investigated the choice of the most effective agents and regimens to ablate superficial tumours and to assess if there is always a place of prophylaxis after successful ablation. It is now timely to consider what has been learnt from these studies.

The first study was a prospective, randomised phase III clinical trial of thiotepa (30 mg weeky for 1 month and monthly for 1 year) versus VM26 (50 mg weekly for 1 month and monthly for 1 year) versus TUR alone in the management of superficial TA, T1 bladder cancer. The aims of the study were to compare the disease-free interval in the three regimens, the recurrence rate and to assess the number of patients with an increase in tumour stage. The disease-free interval was defined as the interval between the initial transurethreal resection and the first biopsy proven recurrence. The recurrence rate was defined as the number of positive cystoscopies divided by the total duration of follow-up. At the conclusion of this study an analysis was carried out of the prognostic factors determining the rate of recurrence and progression in superficial bladder cancer and these prognostic factors were subsequently used to stratify patients in later studies. This study showed that thiotepa was an extremely active agent in the prophylaxis of recurrent bladder tumour, being more active than intravesical VM26 and much more efficient than TUR alone. There was no difference in the time to first recurrence but there was a difference in the subsequent recurrence rate, with thiotepa being the most active prophylactic agent.

The prognostic factor analysis from this study highlighted certain groups of patients who are at a high risk of developing recurrence. These are patients with four or more tumours at presentation, patients who have dysplasia or carcinoma *in situ* in random biopsies of the bladder, patients who have a history of recurrence before their recruitment to the study and patients with tumours of high grade or large size (5 cm or more) (Dalesio *et al.*, 1983).

Following the first study and a number of pilot studies it seemed that the best regimen for intravesical therapy to prevent the recurrence of superficial bladder tumours should involve an intensive 4–6 weeks of treatment followed by monthly prophylaxis for 6 months to 1 year.

The next protocol was for patients with recurrent tumours only and compared intravesical thiotepa (50 mg in 50 ml weekly for 1 month and monthly for 1 year), adriamycin (50 mg in 50 ml weekly for 1 month and monthly for 1 year) and cisplatinum (50 mg in 50 ml weekly for 1 month and monthly for 1 year). It showed adriamycin and thiotepa to be equally effective in the prevention of recurrence of superficial bladder cancer and the recurrence rate in those two arms of the study was the same as the recurrence rate for the thiotepa arm of the first study; the cisplatinum was discontinued because of anaphylactic reactions.

The third study in patients with TA or T1 tumours compared the intravesical adriamycin regimen with intravesical epodyl (1.13 g weekly for 1 month and monthly for 1 year) and the study contained, once more, a third arm of patients who were treated by transurethral resection alone, since in the first study there had been no difference in the three arms with regard to time to first recurrence. This study differed from previous studies in that the end-point was not the time to the first recurrence, but either the first recurrence after 1 year of treatment, or progression. In this way it was hoped to overcome the confusion between residual and truly recurrent tumours and also to give the intravesical agents time to act. Therefore, if patients recurred during the first year they received a further course of treatment following transurethral resection where appropriate, but the total length of treatment did not exceed 12 months.

Half-way through the study, it was quite clear that patients who were receiving intravesical chemotherapy had a very much lower rate of recurrence than those patients in the TUR alone arm and that arm of the study was closed before the entire study had been recruited. The recurrence rates in the two arms, where the patients received intravesical chemotherapy, were statistically inseparable and there appeared to be no difference between the two agents, adriamycin and epodyl, in this situation (Kurth *et al.*, 1985).

The EORTC GU Group was only one group carrying out

Received 12 October 1989; and in revised form 28 October 1989.

such studies throughout the world and at this time it seemed that four agents were equally effective in preventing recurrence of bladder tumours: epodyl, thiotepa, mitomycin C and adriamycin. All these agents had been shown to be effective in ablating superficial bladder tumour in phase II studies and all had been shown to reduce the recurrence of superficial tumours in phase III studies. There were some differences in the toxicity of the regimes and quite a considerable difference in the cost of the agents.

The toxicity of intravesical instillation may be either local or systemic. The local toxicity is chemical or bacterial cystitis and work by Pavone and Jacobi (1982) had suggested that this was more common where patients were treated immediately after resection. Systemic side-effects occur very rarely with the larger molecular weight agents but the smaller molecular weight compounds, such as thiotepa and epodyl, were absorbed following transurethral resections.

In the next two studies we assessed the place of early treatment and clinicians could choose whether they used either mitomycin or adriamycin, and both the agents were to be studied in patients with new or recurrent, single or multiple superficial tumours. The two studies looked at the efficacy and toxicity of early (6 h after TUR) or delayed (7–15 days after TVR) instillation in a prospective manner and also the advantage of intermediate or long-term prophylaxis.

There was a marginal advantage for early instillation with long-term prophylaxis in those patients treated with adriamycin but for mitomycin the time of instillation and the addition of maintenance therapy appear, at this stage, to make little difference. However, early instillation of adriamycin intravesically was associated with a high incidence of chemical cystitis which led to withdrawal from the study of some 4% of patients because of delay or cessation of treatment and its overall advantage was less because of this.

Intravesical instillation of BCG had been used for many years in both the therapeutic and prophylactic treatment of superficial bladder cancer (Morales *et al.*, 1976; Lamm *et al.*, 1980; Brosman, 1982). It had also been shown to be very effective in the therapy of carcinoma *in situ* (Herr *et al.*, 1983). The mode of action of BCG is still not fully understood but it would seem to employ an immunotherapeutic mechanism. BCG has been shown to share some common antigenicity with certain tumour cells. It is a stimulant of the reticulo-endothelial system and of the antibody response, and it increases the delayed hypersensitivity response (Robinson, 1984). By 1984 a number of phase II studies of intravesical BCG in the treatment and prophylaxis of superficial bladder cancer had been completed. There were, however, very few phase III studies and it was therefore decided to mount a phase III study of intravesical BCG (6-weekly instillations to be repeated if recurrence at 3 months) versus intravesical mitomycin C (30 mg in 50 ml saline; month 1, weekly; months 2–6, monthly). The end-point was first recurrence after completion of intravesical treatment. There was, and still is, considerable debate as to what is the most effective strain of BCG but we elected to use BCG RIVM, a strain produced by the Dutch National Institute of Public Health and Environmental Hygiene.

Both intravesical BCG RIVM and intravesical mitomycin C were equally effective in preventing recurrence of superficial bladder tumour. The BCG strain was very much cheaper but intravesical BCG was marginally more toxic locally than mitomycin (Debruyne *et al.*, 1986).

Four studies were also started in 1986, all based on the prognostic factor analysis of earlier studies and two of them attempting to answer a new question. A phase II study of intravesical Connaught BCG for primary or secondary carcinoma *in situ* is in progress. We are also studying whether or not an intravesical agent which is shown to be effective therapeutically is also effective in the same patient as prophylaxis against recurrence. There were two agents the Group were particularly interested in this respect: mitomycin C and 4-epirubicin, a new analogue of the parent adriamcyin compound used in previous studies. The two studies were written as combined phase II/phase III studies. The initial therapy with one of the drugs is administered after all but one lesions in the bladder have been removed, the one being left as a marker lesion. The bladder is examined at 12 weeks, the marker lesion, if it remains, resected or its area biopsied and the patient is then randomised subsequently to receive either maintenance or no maintenance therapy with the same drug.

The patients all have multiple, primary or recurrent tumour and, therefore, should naturally have a high rate of recurrence. The studies are extremely slow to recruit, partly due to some institutions having ethical problems over the leaving of a marker lesion and partly because the studies are naturally quite complex. They have, in fact, already been curtailed and reduced in the number of patients to be studied. Nevertheless, it is hoped that both will ultimately be phase II/phase III studies, although all the objectives may not be achieved. It is hoped that the primary question of the relationship between a therapeutic effect and a prophylactic effect of a give agent administered intravesically can be assessed.

The final study ongoing at the present time is a randomised phase III study in patients with single, new or recurrent TA/T1 tumours who, following transurethral resection, receive a single instillation of 80 mg of 4-epirubicin intravesically. In this study it was decided to re-examine the patient cystoscopically after one month in order to ensure the adequacy of transurethral resection. This study has now recruited over 400 patients and will be closed when a replacement study is ready. This study is investigating, with possibly the most active agent at our disposal, the suggestion first made by Burnard *et al.* (1976) that in patients with a tumour with good prognostic factors a single intravesical instillation is adequate to prevent recurrence.

The EORTC Group has shown that there are a number of active agents which may be used intravesically to prevent recurrence of superficial bladder tumours. These are thiotepa, epodyl, adriamycin, mitomycin, 4-epirubicin and BCG. All these agents are effective in ablative therapy of widely recurrent tumours and many of them, particularly BCG, are effective in the treatment of primary carcinoma *in situ*. While an appropriate regimen with regard to the frequency of instillation seems to have been arrived at for chemoprophylaxis, there are a number of questions with regard to the maximum efficacy of each individual agent in the urine and whether or not buffering or alteration of the constituents of the urine may affect that, which have yet to be studied. There has been no evidence in any of the studies carried out on over 2,000 patients that any of the intravesical agents are effective in preventing progression to a higher stage (Greene *et al.*, 1984; Somerville *et al.*, 1985).

We now feel that the place, mechanism of administration and usefulness of intravesical chemotherapy have been quite clearly defined. The time has come when a more detailed look into the prevention of bladder cancer by removing aetiological agents and by neutralising early pre-cancerous change in cells of the transitional cell urothelium is probably the only way that a major impact is going to be made in reducing the incidence of invasive disease. The complete elimination of tobacco abuse would be a major step forward in the prevention of bladder cancer and more detailed analyses of abnormal urinary constituents in patients who have developed tumours may help to identify other agents which could be removed. It seems likely, for instance, that there are still a number of metabolites of tryptophan and other amino acids that need to be examined in this respect. Stabilisation of the urothelium with retinoids and other vitamin derivatives may be a preventive measure which needs exploring further, and strengtheninig of the aminoglycans layer to prevent urinary constituents from damaging the cell is another possible avenue of research. Early identification of changes in transitional cell epithelium before rapid proliferation of mutated cells may be of benefit and perhaps new therapeutic weapons will be useful against these cells.

The careful and sensible use of intravesical chemotherapy is probably only the tip of the iceberg in our efforts to conquer bladder cancer. It has its role in a clearly defined group of patients, it can be simply administered in ordinary district hospitals on a day case basis and the toxicity is minimal although the expense is not. It is, however, sad to relate that for all our efforts the mortality from bladder cancer is still high and we seem still incapable of preventing the emergence of invasive tumours by the present methods available to us.

I acknowledge with great pleasure and gratitude the efficiency and endeavour of the following who were the co-ordinators of the studies described in this article: Professor C. Schulman, Brussels; Mr M.R.G. Robinson, Pontefract; Professor L. Denis, Antwerp; Professor K.H. Kurth, Amsterdam; Dr C. Bouffioux, Liege; Professor F. Debruyne, Nijmegen; Dr G. Jakse, Munich; Dr W. Oosterlinck, Gent; Professor A. Bono, Varese; Mr R. Hall, Newcastle upon Tyne.

### References

BURNARD, K.G., BOYD, P.J.R., MAYO, M.E., SHUTTLEWORTH, K.E.D. & LLOYD-DAVIES, R.W. (1976). Single dose intravesical Thiotepa as an adjuvant to cystodiathermy in the treatment of transitional cell bladder carcinoma. *Br. J. Urol.*, **48**, 55.

BROSMAN, S.A. (1982). Experience with BCG in patients with superficial bladder cancer. *J. Urol.*, **128**, 27.

DALESIO, O., SCHULMAN, C.C., SYLVESTER, R. & 6 others (1983). Prognostic factors in superficial bladder tumours – a study of the European Organisation for Research and Treatmen of Cancer Genito-Urinary Tract Cancer Group. *J. Urol.*, **129**, 730.

DEBRUYNE, F., VAN DER MEYDEN, A.P.M., SCHREINECRES, L.M.H. & 6 others (1988). BCG RIVM intravesical immunoprophylaxis for superficial bladder cancer. In *Progress and Controversies in Oncological Urology II, EORTC GU Group Monograph No. V*, Schroder, F.H., Klijn, J.G.M., Kurth, K.H., Pinedo, H.M., Splinter, T.A.W. & De Voogt, H.J. (eds) p. 511. Alan R. Liss: New York.

DENIS, L. (1983). Anaphylactic reactions to repeated intravesical instillations of Cisplatinum – letter to the Editor. *Lancet*, **i**, 1378.

DENIS, L., BOUFFIOUX, C., KURTH, K.H. & 4 others (1987). Current status of intravesical chemotherapy trials in the EORTC Urological Group. An overview. *Cancer Chemother. Pharmacol.*, **20** (suppl.), 67.

GREENE, L.F., HANASH, K.A. & FARROW, G.M. (1973). Benign papilloma or papillary carcinoma of the bladder? *J. Urol.*, **110**, 205.

GREENE, D.F., ROBINSON, M.R.G., GLASHAN, R, NEWLING, D.W.W., DALESIO, O. & SMITH, P.H. (1984). Does intravesical chemotherapy prevent invasive bladder cancer? *J. Urol.*, **131**, 33.

HERR, H.W., PINSKY, C.M., WHITMORE, W.F. Jr, OETTGEN, H.F. & MELAMED, M.R. (1983). Effective intravesical BCG on carcinoma in situ of the bladder. *Cancer*, **51**, 132.

JACOBI, G. (1982). Chemotherapy for urinary bladder cancer: developments, trends and future preventitives. In *Clinical Bladder Cancer*, Denis, L., Smith, P. & Pavone Macaluso, M. (eds) p. 93 Plenum Press: New York.

KURTH, K.H., TUNN, A., AY, R. & 6 others (1984). Adjuvant chemotherapy in superficial transitional cell bladder carcinoma – an EORTC randomized trial comparing doxyrubicin hydrochloride ethoglucid and TUR alone. *J. Urol.*, **132**, 258.

LAMM, D.L., THOR, D.E., HARRIS, S.C., REYNA, J.A., STOGDILL, V.D. & RADWIN, H.M. (1980). BCG immunotherapy with superficial bladder cancer. *J. Urol.*, **124**, 38.

LERMAN, R.I., HUTTER, R.V. & WHITMORE, W.F. Jr. (1970). Papilloma of the urinary bladder. *Cancer*, **25**, 333.

MISHINA, T., ODA, K., MURATA, S., OOE, H., MORI, Y. & TAKAHASKI, T. (1975). Mitomycin C bladder instillation therapy for bladder tumours. *J. Urol.*, **114**, 217.

MORALES, A., EIDINGER, D. & BRUCE, A.W. (1976). Intracavity bacillus Calmette-Guerin in the treatment of superficial bladder tumours. *J. Urol.*, **116**, 180.

RIDDLE, P.R. (1973). The management of superficial bladder tumours with intravesical epodyl. *Br. J. Urol.*, **45**, 84.

ROBINSON, M.R.G. (1984). BCG in the management of superficial bladder cancer. In *EORTC GU Group Monograph No. 2 Part B. Superficial Bladder Tumours*, Schroder, F.H. & Richards, B. (eds) p. 161. Alan R Liss: New York.

SCHULMAN, C., ROZENCWEIG, M., STAQUET, M., KENIS, Y. & SYLVESTER, R. (1976). EORTC randomized for trial for the adjuvant therapy of T1 bladder carcinoma. *Eur. Urol.*, **2**, 271.

SOMERVILLE, J.J.F., NEWLING, D.W.W., RICHARDS, B., ROBINSON, M.R.G. & SMITH, P.H. (1985). Mitomycin C in superficial bladder cancer – 24 month follow up. *Br. J. Urol.*, **57**, 686.